# The Basal NPO *crh* Fluctuation is Sustained Under Compromised Glucocorticoid Signaling in Diurnal Zebrafish

**DOI:** 10.3389/fnins.2015.00436

**Published:** 2015-12-02

**Authors:** Chen-Min Yeh

**Affiliations:** Developmental Genetics of the Nervous System, Max Planck Institute for Medical ResearchHeidelberg, Germany

**Keywords:** the hypothalamo-pituitary-adrenal/interrenal axis, neurosecretory preoptic area, cortisol, corticortropin-releasing hormone, circadian variation, negative feedback, diurnal zebrafish larva

## Abstract

The circadian activity of the hypothalamo-pituitary-adrenal/interrenal (HPA/I) axis is crucial for maintaining vertebrate homeostasis. In mammals, both the principle regulator, corticotropin-releasing hormone (*crh*) in the hypothalamic paraventricular nucleus (PVN) and the final effector, the glucocorticoids show daily rhythmic patterns. While glucocorticoids are the main negative regulator of PVN *crh* under stress, whether they modulate the PVN *crh* rhythm under basal condition is unclear in diurnal animals. Using zebrafish larvae, a recently-established diurnal model organism suited for the HPA/I axis and homeostasis research, we ask if glucocorticoid changes are required to maintain the daily variation of PVN *crh*. We first characterized the development of the HPI axis overtime and showed that the basal activity of the HPI axis is robust and tightly regulated by circadian cue in 6-day old larvae. We demonstrated a negative correlation between the basal cortisol and neurosecretory preoptic area (NPO) *crh* variations. To test if cortisol drives NPO *crh* variation, we analyzed the NPO *crh* levels in glucorcorticoid antagonist-treated larvae and mutants lacking circadian cortisol variations. We showed that NPO *crh* basal fluctuation is sustained although the level was decreased without proper cortisol signaling in zebrafish. Our data indicates that glucocorticoids do not modulate the basal NPO *crh* variations but may be required for maintaining overall NPO *crh* levels. This further suggests that under basal and stress conditions the HPA/I axis activity is modulated differently by glucocorticoids.

## Introduction

The neuroendocrine system hypothalamo-pituitary-adrenal/interrenal (HPA/I) axis plays an essential role in maintaining the homeostasis of vertebrates under fluctuating environment (Charmandari et al., 2005; Chrousos, 2009). To regulate body physiology under both basal and stress conditions, the activity of HPA/I axis components were tightly linked to each other and subjected to external stimuli (Tsigos and Chrousos, 2002). Until now, the basal circadian and stress-induced variations of the HPA axis and of its final effectors glucocorticoids have been well characterized particularly in rodents (Watts, 1996; Watts et al., 2004; de Kloet et al., 2005). It is known that corticortropin-releasing hormone (CRH) from the hypothalamic paraventricular nucleus (PVN) and adrenocorticotropic hormone (ACTH) from the pituitary play essential roles in regulating glucocorticoid variations (Muglia et al., 1997; Smith et al., 1998; Liu et al., 2011) and glucocorticoids negatively modulate these factors through genomic or non-genomic mechanisms (Malkoski and Dorin, 1999; Newton, 2000; Herman et al., 2012).

Under stress, glucocorticoids negatively regulate PVN *crh* transcripts through a glucocorticoid receptor-dependent mechanism (Malkoski and Dorin, 1999; van Der Laan et al., 2009; Jeanneteau et al., 2012). However, whether under basal condition glucocorticoids play a role in generating or maintaining the circadian pattern of PVN *crh in vivo* is not clear for diurnal animals. Although, the level of PVN *crh* transcripts negatively correlates with the circadian range of corticosterone (main glucocorticoids in rodent), diminishing the circadian variation of corticosterone or the glucorcoticoid signaling did not eliminate the basal rhythm of PVN *crh* transcript in rat and mice (Watts et al., 2004; Laryea et al., 2013, 2015). This suggests that at least in nocturnal rodents, glucocorticoid-mediated negative feedback is not required for the circadian PVN *crh* variations.

The zebrafish, *Danio rerio* is considered as a suited vertebrate model for studying the neuroendocrine system of diurnal animals. Zebrafish HPI axis shares conserved anatomical, molecular and functional features with the HPA axis of mammals (To et al., 2007; Alsop and Vijayan, 2009; Löhr and Hammerschmidt, 2011). The hypothalamus and pituitary of zebrafish process the conserved signaling molecules and cell types (Liu et al., 2003; Dickmeis et al., 2007; Herget et al., 2014). The larval stage of zebrafish has been used to understand HPA axis- and glucocorticoid-related physiology and behavior (Clark et al., 2011; Steenbergen et al., 2011). At 5 days old, zebrafish larvae show robust increases of cortisol level upon stress exposures (Yeh et al., 2013). Alterations in the glucocorticoid signaling induce specific cellular circadian and metabolic defects (Dickmeis et al., 2007; Lin et al., 2011). It also disrupts normal development and changes stress-related behaviors in larvae and adults (Griffiths et al., 2012; Nesan et al., 2012; Ziv et al., 2013). Yet, the circadian-related characteristics of the HPI axis are largely unknown in this diurnal organism.

Therefore, here the circadian activity of the HPI axis and the role of glucocorticoids in modulating basal HPI axis activity were addressed. We first characterized the circadian patterns of the HPI axis in developing zebrafish larvae. We showed that the HPI axis is fully mature and tightly regulated through circadian light cues in 6 day-old larvae. We observed the negative correlation between the basal cortisol and NPO *crh* suggesting the presence of the glucocorticoid-mediated negative feedback. We then tested if cortisol modulates the NPO *crh* daily variation using larvae with compromised circadian cortisol variation and cortisol signaling. Our results indicate that the basal variation of NPO *crh* transcripts in zebrafish is maintained in a glucocorticoid-independent manner although the level is decreased. Under basal condition, glucocorticoids is likely to play a critical role in regulating the overall level of NPO *crh* rather than sustaining its fluctuation.

## Materials and methods

### Zebrafish maintenance, treatment and strains

Zebrafish breeding and maintenance was performed under standard conditions (Westerfield, 2000). Embryos were collected in the morning and raised on a 12:12 light/dark cycle in E2 medium or E2 medium with 0.2 mM 1-phenyl-2-thiourea to avoid pigment formation (Westerfield, 2000). Larvae were incubated from 5 dpf evening in 2 μM Mifepristone (RU-486, Sigma-Aldrich) dissolved in E2-Medium with 0.1% DMSO (Weger et al., 2012). AB/TL wild type strain is used. *Rx3* fishes were incrossed and their progenies were screened for the presence of eyes for homozygous at 2 dpf (Dickmeis et al., 2007). The experiments were performed on 6 dpf larvae unless further indicated. Zebrafish experimental procedures were performed according to the guidelines of the German animal welfare law and approved by the animal protection office of the Max Planck Institute and the regional government office of Karlsruhe.

### Cortisol ELISA

Groups of 30 larvae were immobilized in ice water, frozen in ethanol/dry ice bath, and stored at −20°C. Cortisol from homogenized samples was extracted with ethyl acetate. We employed the extraction and cortisol ELISA protocol (Yeh et al., 2013) using cortisol mouse antibody (EastCoast Bio), cortisol standards (Hydrocortisone, Sigma-Aldrich) and Cortisol-HRP (EastCoast Bio). The reactions were stopped using 1M sulfuric acid and read at 450 nm in an ELISA-reader (Multiskan Ascent, Thermo Scientific). The data were corrected for dilution factor, extraction efficiency and recovery function.

### Probes, *in situ* hybridization (ISH) and image analysis

Whole-mount ISH was performed as described (Hauptmann and Gerster, 1994). The design of *crh* in situ probe is described previously (Löhr et al., 2009). To quantify cell numbers, the trunks of the larvae were cut off to avoid orientation problem. The cells were visualized from dorsal view under the DIC microscope using 10x objective lens (Leica, DM5500).

### qRT-PCR

Total RNA was extracted from 30 larvae (*N* = 4–5) using Trizol (Invitrogen) and PureLink RNA Mini Kit (Ambion). qRT-PCR was performed using Power SYBR® Green RNA-to-C_*T*_™ 1-Step kit (Applied Biosystems) with Applied Biosystems 7500 RT-PCR system. Primers: *ef1alpha* mRNA_Forward: CTGGAGGCCAGCTCAAACGT; Reverse:ATCAAGAAGAGTAGTACCGCTAGCATTAC. *Period circadian clock, per4* (Cavallari et al., 2011).

### Statistical analysis

All data are shown as mean and standard error of the mean (S.E.M.). We used Student's *t*-tests (two-tailed) for two-group comparisons, or Mann-Whitney *U*-tests if the data did not fulfill the assumptions of the *t*-test. We used ANOVAs for multiple group comparisons, followed by Bonferroni's *post-hoc* tests. We analyzed data using Prism 5 (Graphpad Software). ^*^ indicates *p* < 0.05, ^**^ indicates *p* < 0.01 and ^***^ indicates *p* < 0.001.

### Experimental design

Cortisol circadian fluctuation in larvae was determined by three steps. First, the development of cortisol pattern is determined by measuring cortisol levels from 2 dpf evening to 6 dpf evening (*N* = 12–18). To address if cortisol fluctuations are driven by the internal circadian clock, larvae were raised under light/dark cycle until 4 dpf and kept under darkness until being sampled for cortisol levels (*N* = 9–15). Finally, to address if light establishes cortisol circadian fluctuations, the larvae were raised under darkness until being sampled (*N* = 12).

To investigate the relationship between basal NPO *crh* and cortisol level, we analyzed the daily variation of NPO *crh* mRNA positive cells (examples shown in **Figures 2A–D**; refer as NPO *crh* in the following text) in 6 dpf larvae when the cortisol fluctuation is robust (*N* = 23–24). Miprefistone (MIF), an antagonist of glucocorticoids that binds to GRs and diminishes the glucocorticoid signaling (*N* = 19–37; Andrews et al., 2012; Weger et al., 2012) and *rx3* mutants whose cortisol signaling has been proved to be defective (cortisol: *N* = 6–10; *crh: N* = 12–15; Dickmeis et al., 2007) were used to addressed the effects of cortisol on NPO *crh*. The results were reported from one of two people who counted the NPO *crh* cell number blindly. One experiment, the MIF treated experiment is repeated and the result is confirmed by one additional person.

## Result

### The establishment of the HPI axis circadian activity in zebrafish larvae

**Figure 1:**
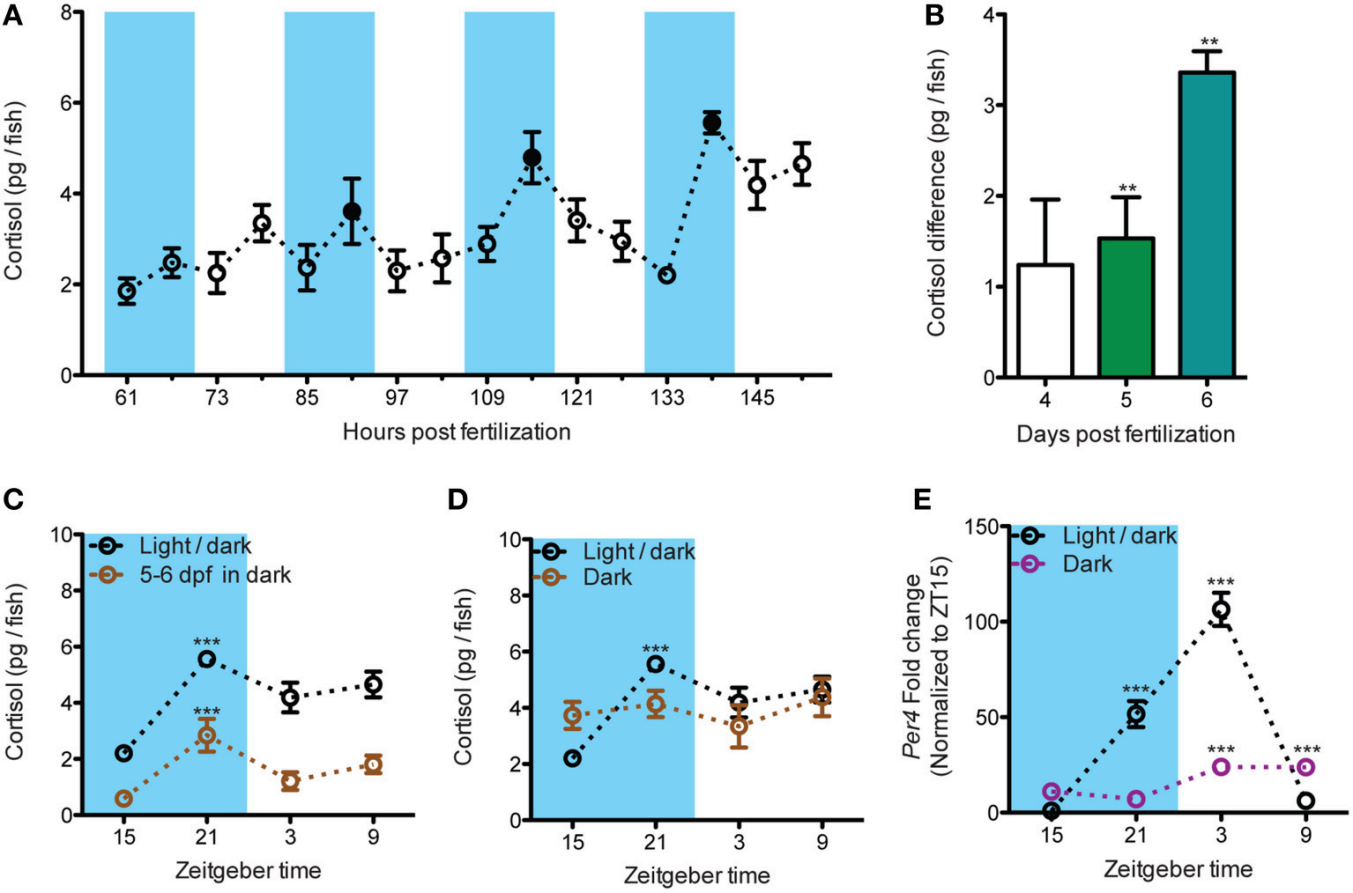
**The establishment of the circadian cortisol rhythm during early larval development. (A)** Establishment of circadian cortisol rhythm under light/dark cycles in developing larvae (dark periods are shadowed in blue). A repeated increase of cortisol level can be seen at each dark cycles starting from 85 hpf. The daily peak of cortisol was indicated by the black spots (*N*_61−103hpf_ = 18, *N*_109−151hpf_ = 12). **(B)** The amplitude of daily cortisol fluctuation during dark phases increased overtime. The amplitude was calculated from the cortisol difference at each dark period (4 dpf: 91 hpf - 85 hpf, 5 dpf: 115 hpf - 109 hpf, and 6 dpf: 139 hpf - 133 hpf; *N*_4dpf_ and *N*_5dpf_ = 18, *N*_6dpf_ = 12). **(C)** The circadian fluctuation of cortisol is maintained in larvae deprived from light cue for 24 h (*N* = 9–15). **(D)** The circadian cortisol fluctuation is blunted in larvae raised without circadian light cue (*N* = 12). **(E)** *per4* mRNA circadian fluctuation is altered in larvae raised without circadian light cue (*N* = 4–5). ^**^p < 0.01 and ^***^p < 0.001.

The measurement of basal cortisol level every 6 hours from 61 to 151 hours post fertilization (hpf) showed that, starting from 85 hpf, a repeated daily fluctuation of cortisol can be detected. The nadir of cortisol level occurred at ZT15 (zeitgeber time 15, 11 p.m.) and the level increased to the peak at ZT21 (5 a.m.; Figure 1A: black dots). The differences of cortisol level between these two continuous time points became larger over days and are differing from 0 starting at 5 dpf (Figure 1B: Wilcoxon Signed Rank Test: 4 dpf: *p* = 0.107; 5 dpf: *p* = 0.003; 6 dpf: *p* = 0.003). Furthermore, larvae raised under circadian light cue until 4 dpf were able to maintain the cortisol fluctuation under darkness at 6 dpf (Figure 1C: One-way ANOVA, Light/dark: *F* = 14.2, *p* < 0.001; 5–6 dpf dark: *F* = 6.7, *p* < 0.001, Bonferroni's post-tests: compare all pairs of columns and significant difference from the previous time point are shown). Noteworthy, larvae never exposed to circadian light cue did not establish proper cortisol basal variations (Figure 1D: One-way ANOVA, Dark: *F* = 0.6, *p* = 0.642). The dampening of the cortisol rhythm is accompanied by the alteration of the core circadian regulating gene, *period circadian clock, per4* [Figure 1E: Two-way ANOVA, treatment × time: *F*_(3, 28)_ = 81.5, *p* < 0.001; Bonferroni's post-tests for significant difference from ZT 15 group]. In sum, we showed a robust circadian rhythm of cortisol which is established overtime in the developing zebrafish larvae by the circadian light cues.

### Negative correlations between NPO *crh* number and cortisol level under basal condition

**Figure 2:**
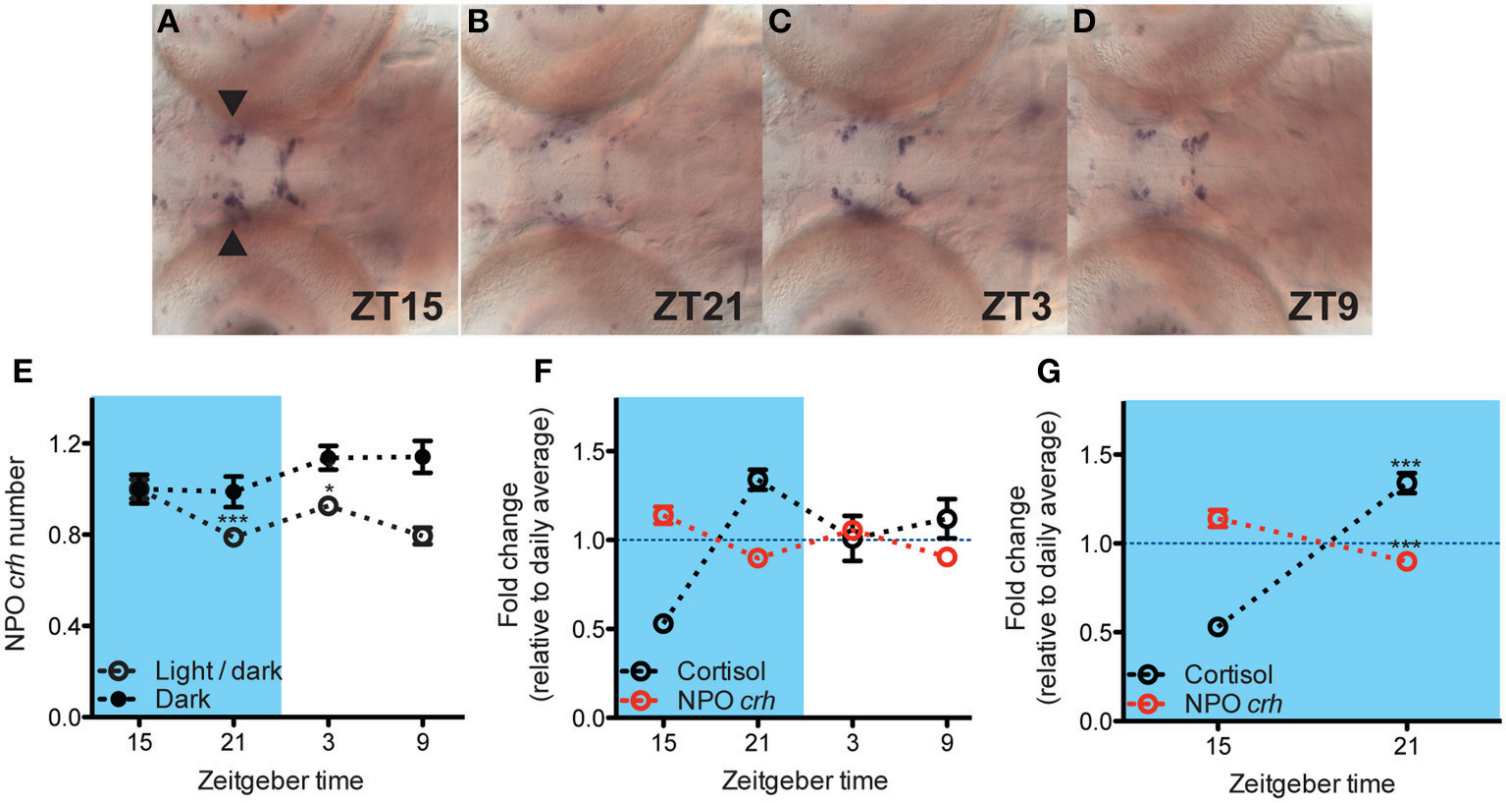
**Negative correlation between NPO *crh* and cortisol level under basal condition**. **(A–D)** Representative *crh* in situ pictures at 4 different time points. NPO *crh* mRNA positive cells (arrowhead) at different time were quantified. **(E)** The NPO *crh* shows a daily variation which is abolished by light deprivation (*N* = 23–24) **(F)** Daily variations of NPO *crh* and cortisol level showed reversed patterns. NPO *crh* and cortisol were plotted together after normalized to their daily average which was set to 1 respectively (Cortisol: *N* = 12; NPO *crh*: *N* = 23–24). **(G)** Negative correlation can be detected between NPO *crh* cell number and cortisol level when cortisol rises from its nadir to peak level (data from **G**). ^*^p < 0.05 and ^***^p < 0.001.

Next, we addressed the relationship between cortisol and NPO *crh*. Under basal condition, NPO *crh* levels show a daily variation which is eliminated in larvae deprived from circadian light cue (Figures 2A–E: One-way ANOVA, Light/dark: *F* = 8.6, *p* < 0.001; Dark: *F* = 1.8, *p* = 0.1598, Bonferroni's post-tests: compare all pairs of columns and significant difference from the previous time point are shown). As predicted from previous studies in rodent, we observed that the variation of NPO *crh* corresponded to that of cortisol in an inverse manner (Figure 2F). Importantly, from ZT15 to ZT21 when cortisol rises from its nadir to its peak, there is a corresponding decrease of NPO *crh* [Figure 2G: Two-way ANOVA, treatment × time: *F*_(1, 67)_ = 117.9, *p* < 0.001; Bonferroni's post-tests for significant difference from ZT 15 group]. In summary, the NPO *crh* displays a variation regulated by circadian light cues and correlates negatively to the cortisol rhythms.

### NPO *crh* daily fluctuations and cortisol signaling

**Figure 3:**
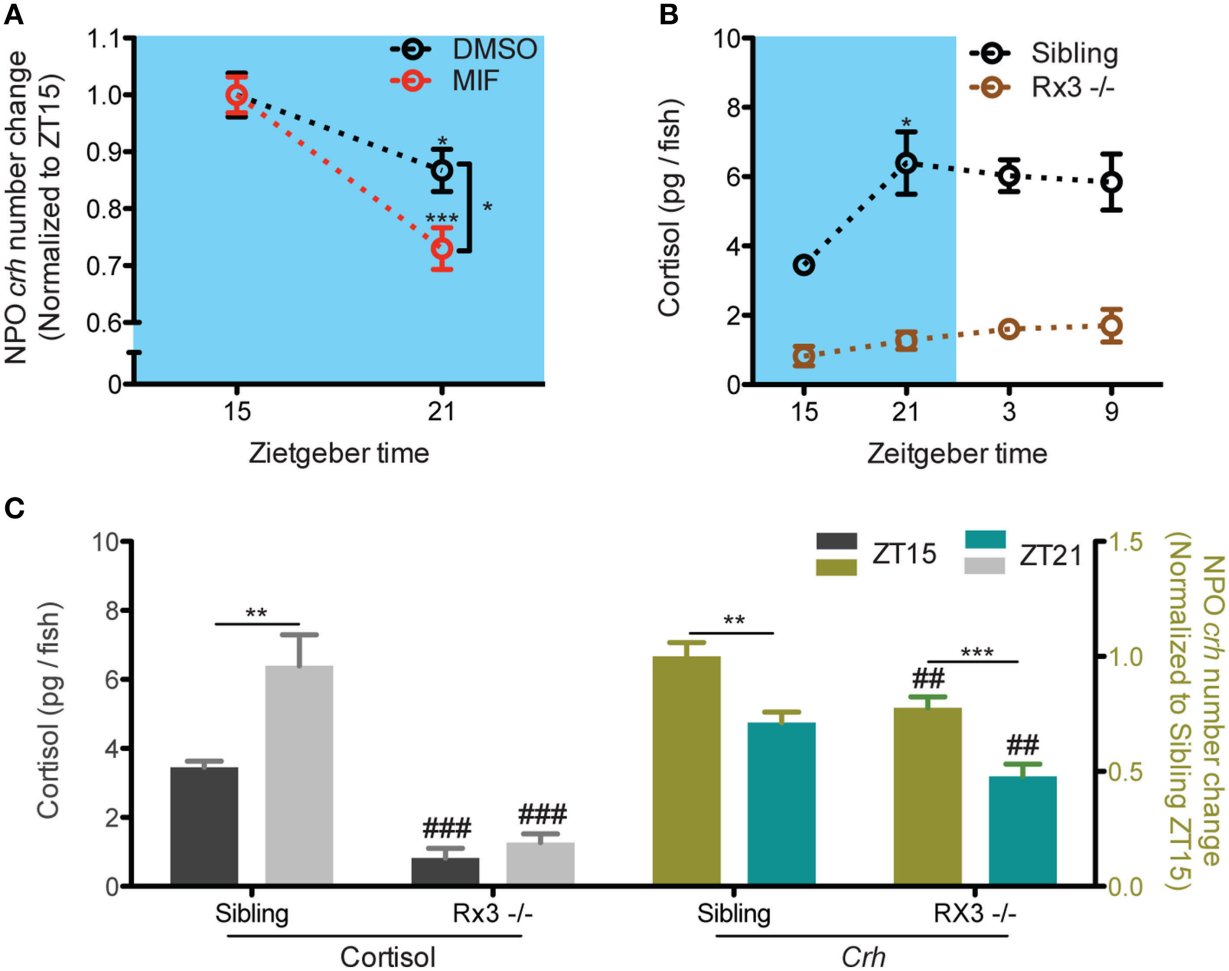
**NPO *Crh* variation but not the level was sustained in larvae with compromised cortisol signaling. (A)** MIF did not alter the fluctuation of NPO *crh* from ZT15 to ZT21 but changed the level of NPO *crh at* ZT21 (DMSO: *N*_*ZT*15_ = 19, *N*_*ZT*21_ = 19; MIF: *N*_*ZT*15_ = 37, *N*_*ZT*21_ = 20). **(B)** No cortisol rhythm can be detected in *rx3* mutant in contrast to that in their siblings (*Rx3* sibling: *N* = 6; *rx3*: *N* = 10). **(C)** Normal NPO *crh* but not cortisol fluctuation can be detected between from ZT15 to ZT21 in *rx3* mutant. The levels of NPO *crh* and cortisol are reduced in *rx3* mutants (Cortisol: *rx3* sibling: *N* = 6; *rx3*: *N* = 10; *crh*: *rx3* sibling: *N*_*ZT*15_ = 14 and *N*_*ZT*21_ = 12; *rx3*: *N* = 15). ^*^p < 0.05, ^**^^, *##*^*p* < 0.01 and, ^***^^, *###*^*p* < 0.001.

Next, we directly addressed if the negative feedback of cortisol results in NPO *crh* decrease. The reduction of NPO *crh* from ZT15 to ZT21 was still observed under overnight miprefistone (MIF) treatment (Figure 3A: *t*-test, DMSO: *T* = 2.5, *p* = 0.018; MIF: *T* = 5.4, *p* < 0.001, for significant difference from ZT21 group). The level of NPO *crh* at ZT21 is lower with MIF treatment (Figure 3A: *t*-test, *T* = 2.6, *p* = 0.012). Furthermore, we used *rx3* mutants which showed undetectable circadian cortisol fluctuation (Figure 3B: One-way ANOVA, Sibling: *F* = 4.2, *p* = 0.0188; Rx3 -/-: *F* = 1.6, *p* = 0.2154, Bonferroni's post-tests: compare all pairs of columns and significant difference from the previous time point are shown). In the mutant larvae, the decrease of NPO *crh* from ZT 15 to ZT 21 was sustained (Figure 3C: *t*-test, sibling cortisol: *T* = 3.2, *p* = 0.009; *rx3* cortisol: *T* = 1.2, *p* = 0.245; sibling *crh*:*T* = 3.7, *p* = 0.001; *rx3 crh*: *T* = 4.2, *p* < 0.001). The levels of cortisol and NPO *crh* are lower in *rx3* comparing with their siblings (Figure 3A: *t*-test, cortisol ZT15: *T* = 6.8, *p* < 0.001; cortisol ZT21: *T* = 6.8, *p* < 0.001; *crh* ZT15: *T* = 2.9, *p* = 0.007; *crh* ZT21: *T* = 3.2, *p* = 0.003). Thus, our data suggests that under basal condition, the decrease of NPO *crh* is not driven by the negative feedback of cortisol signaling. Nevertheless, cortisol may be required to maintain proper basal NPO *crh* levels.

## Discussion

In this study, the circadian cortisol variations and its link to the NPO *crh* changes were addressed using the diurnal zebrafish larvae. As the first step, we reported the maturation of the HPI axis and found robust circadian rhythms of cortisol and NPO *crh* daily variation in 6 dpf larvae. We showed a negative correlation between NPO *crh* and cortisol indicating the presence of glucocorticoid-mediated negative feedback. Interestingly, diminishing cortisol signaling using glucocorticoid antagonist and mutants does not abolish the NPO *crh* daily variations but alter the levels of NPO *crh*. This suggests that the basal fluctuation but not the level of the hypothalamic *crh* is independent of the proper circadian glucocorticoid signaling in diurnal organisms.

### The circadian activity of the HPI axis in developing zebrafish larvae

Zebrafish larva is considered as a suited model to understand homeostasis and stress regulation for diurnal animals (Alderman and Bernier, 2009; Alsop and Vijayan, 2009; Nesan et al., 2012; Ziv et al., 2013). Yet, the circadian cortisol pattern and HPI axis activity in this model are unknown. Here we showed that the circadian rhythm of cortisol is established overtime and controlled by circadian environmental light cue. The increase of cortisol observed from late evening to early morning corresponds to the glucocorticoid circadian increase in human overnight and to the circadian fluctuation in rodents (Watts et al., 2004; Dimitrov et al., 2009). The sustained cortisol variation in larvae transferred into darkness (Figure 1C) further indicates that the cortisol rhythm is maintained by the internal circadian clock system. We note that while the cortisol fluctuation is abolished in light-deprived larvae (Figure 1D), the average daily cortisol levels in normal vs. light-deprived larvae do not differ from each other (data not shown). This suggests that light deprivation does not alter the normal development of the HPI axis.

Zebrafish larva at the stage of 6 dpf could be a suited model to study circadian-related properties of the HPA/I axis. Our data strongly suggest that the HPI axis activity in these larvae is tightly regulated by the intrinsic circadian clock. The circadian fluctuation of cortisol and the decrease of NPO *crh* from the late evening to early morning are robust and consistently observed in different genotypes including AB stain of wildtype, *rx3* siblings and *rx3* mutants in our work. In addition, the fact that these larvae responses to different environmental stimuli in a dose-dependent manner also supports the notion that the functionality of the HPI axis is well established (Yeh et al., 2013). This model could be further used to understand the circadian interactions between the HPI axis and other physiological parameters as it has been shown that the physiology and behaviors of 5–6 dpf larvae are also regulated under tight circadian clock control (Kazimi and Cahill, 1999; Whitmore et al., 2000; Hurd and Cahill, 2002; Cavallari et al., 2011).

### NPO *crh* fluctuation was sustained when cortisol signaling was diminished

A prominent function of glucocorticoids is to negatively regulate the upstream HPA axis components (Keller-Wood and Dallman, 1984; Kovács et al., 2000; Herman et al., 2012). The negative correlations between cortisol and NPO *crh* in this study were consistent with data from adult mammals (Watts, 1996; Watts et al., 2004). Our results from *rx3* mutants and MIF treated larvae suggest that robust glucocorticoid fluctuation is not necessary to maintain NPO *crh* basal variation. This coincided with the studies in rodent in which PVN *crh* transcript variation is maintained after adrenalectomy both under basal and stress conditions (Watts et al., 2004; Shepard et al., 2005). The basal rhythm of NPO *crh* could be primarily driven by the SCN since the anatomical connection and functional interactions between the SCN and PVN were well documented in mammals (Moore and Eichler, 1972; Watts and Swanson, 1987; Watts et al., 1987; Buijs et al., 1993; Kalsbeek et al., 2012).

While the circadian cortisol increase did not drive the NPO *crh* decrease, the negative feedback from glucocorticoids to NPO *crh* has been suggested in zebrafish. We observed NPO *crh* decrease using dexamethasone treatment (data not shown) and others have shown it in adult zebrafish (Ziv et al., 2013). We observed that cortisol modulates the overall level but not the variation of the NPO *crh* under basal condition. It is worth to note that more works are needed to rigorously address if proper cortisol signaling is required to maintain the overall NPO *crh* level as our data from *rx3* mutants is subjected to the function of *rx3* on the forebrain development (Stigloher et al., 2006). Also, to understand how NPO *crh* is modulated using larval zebrafish, the connection between NPO and other regions of the brain including the the homologous structures of mammalian hippocampus and amygdala should be explored in this model organism (Tsigos and Chrousos, 2002).

In conclusion, we showed that the HPI axis is fully mature and tightly regulated through circadian light cues in 6 day-old larvae. We showed that the circadian-related properties of the HPI axis in zebrafish are shared with those of the HPA aixs in mammals. We showed that the robust daily NPO *crh* fluctuation can be maintained but the level of NPO *crh* is altered when the cortisol signaling is compromised. Thus, although the basal glucocorticoids change is correlated with the hypothalamic *crh* variation, the negative feedback of the HPI/HPA axis is not driving the hypothalamic *crh* basal variation in the diurnal zebrafish.

## Funding

This work was supported by the Max Planck Society.

### Conflict of interest statement

The author declares that the research was conducted in the absence of any commercial or financial relationships that could be construed as a potential conflict of interest.
